# Development and analytical validation of AB40, AB42, A‐Syn, CD33, P‐Tau 181, Tau, and TREM2 in human serum, plasma, or CSF

**DOI:** 10.1002/alz.089257

**Published:** 2025-01-09

**Authors:** Jacqueline Surls, Robyn Vega Ibanez, Lindsey Brown, Josh Kemp

**Affiliations:** ^1^ Rules‐Based Medicine, Inc, Austin, TX USA

## Abstract

**Background:**

Measurement of blood‐based biomarkers for Alzheimer disease is simpler and more accessible when compared to CSF. We report the development and validation of seven Simoa® immunoassays for the detection of AB40, AB42, A‐Syn, CD33, P‐Tau 181, Tau, and TREM2 in human serum, plasma, or CSF.

**Method:**

Using the Quanterix® Simoa® technology (SR‐X) platform, AB40, AB42, A‐Syn, CD33, P‐Tau 181, Tau, and TREM2 were developed and analytically validated in human serum, plasma, or CSF per CLSI standards.

**Result:**

We have developed and analytically validated Simoa® immunoassays to detect AB40, AB42, A‐Syn, CD33, P‐Tau 181, Tau, and TREM2 in human serum, plasma, or CSF. The assays met acceptance criteria for sensitivity, precision, stability, interference, and parallelism. The calibration curves in serum/plasma were linear from 16‐2000 pg/mL for AB40, 18‐40,000 pg/mL for AB42, 0.13‐80 ng/mL for A‐Syn, 0.012‐60 ng/mL for CD33, 0.60‐3000 pg/mL for P‐Tau 181, 0.12‐600 pg/mL for Tau, and 0.30‐38 ng/mL for TREM2. For CSF, the linear ranges were from 626‐80,000 pg/mL for AB40, 72‐160,000 pg/mL for AB42, 0.013‐8.0 ng/mL for A‐Syn, 0.012‐60 ng/mL for CD33, 30‐150,000 pg/mL for P‐Tau 181, 0.30‐1500 pg/mL for Tau, and 0.60‐76 ng/mL for TREM2. The three quality controls met inter assay precision with %CV ≤16% for levels 1 and 2, and ≤20% for level 3 across all analytes. No interference was observed with bilirubin, hemoglobin, or triglyceride. The sensitivity of the assays in human serum/plasma was demonstrated by the LLOQ values of 28 pg/mL, 47 pg/mL, 0.20 ng/mL, 0.11 ng/mL, 2.8 pg/mL, 0.22 pg/mL, and 0.59 ng/mL for AB40, AB42, A‐Syn, CD33, P‐Tau 181, Tau, and TREM2, respectively. For human CSF, the LLOQ values of 1139 pg/mL, 187 pg/mL, 0.020 ng/mL, 0.11 ng/mL, 142 pg/mL, 0.56 pg/mL, and 1.2 ng/mL for AB40, AB42, A‐Syn, CD33, P‐Tau 181, Tau, and TREM2 in human CSF, respectively. The stability of the analytes was established under various conditions, such as freeze‐thaw cycles, short‐term and long‐term storage.

**Conclusion:**

These assays demonstrated acceptable performance for clinical implementation as research use only and may be useful for blood‐based biomarker studies in clinical trials.

